# Niclosamide Induces Cell Cycle Arrest in G1 Phase in Head and Neck Squamous Cell Carcinoma Through Let-7d/CDC34 Axis

**DOI:** 10.3389/fphar.2018.01544

**Published:** 2019-01-09

**Authors:** Zewen Han, Qingxiang Li, Yifei Wang, Lin Wang, Xiaoxu Li, Na Ge, Yixiang Wang, Chuanbin Guo

**Affiliations:** ^1^Department of Oral and Maxillofacial Surgery, Peking University School and Hospital of Stomatology, Beijing, China; ^2^Central Laboratory, Peking University School and Hospital of Stomatology, Beijing, China

**Keywords:** niclosamide, cell cycle, G1 arrest, let-7d/CDC34 axis, anti-cancer

## Abstract

Niclosamide is a traditional anti-tapeworm drug that exhibits potent anti-cancer activity. Our previous study showed that niclosamide induces cell cycle arrest in G1 phase. Nevertheless, the underlying mechanism remains unknown. The following study investigated the molecular mechanism through which niclosamide induced G1 arrest in head and neck squamous cell carcinoma (HNSCC) cell lines. The effect of niclosamide on human HNSCC cell line WSU-HN6 and CNE-2Z were analyzed using IncuCyte ZOOM^TM^ assay, flow cytometry (FCM), real-time PCR and western blot. Luciferase assay was conducted to demonstrate the interaction between let-7d (a let-7 family member which functions as a tumor suppressor by regulating cell cycle) and 3′UTR of CDC34 mRNA. Xenografts tumor model was established to evaluate the niclosamide treatment efficacy *in vivo*. Briefly, an exposure to niclosamide treatment led to an increased let-7d expression and a decreased expression of cell cycle regulator CDC34, finally leading to G1 phase arrest. Moreover, an overexpression of let-7d induced G1 phase arrest and downregulated CDC34, while the knockdown of let-7d partially rescued the niclosamide-induced G1 phase arrest. Luciferase assay confirmed the direct inhibition of CDC34 through the targeting of let-7d. Furthermore, niclosamide markedly inhibited the xenografts growth through up-regulation of let-7d and down-regulation of CDC34. To sum up, our findings suggest that niclosamide induces cell cycle arrest in G1 phase in HNSCC through let-7d/CDC34 axis, which enriches the anti-cancer mechanism of niclosamide.

## Introduction

Head and neck squamous cell carcinoma (HNSCC) is a common disease found in the head and neck region (Siegel et al., 2017). The 5 years survival for HNSCC is lower than 60%, which is inconsistent with the great advances in treatment approach, including surgery, digital guided technology, radio-, chemo-, and targeted immunotherapy (Chang and Wang, 2016). To achieve better management of HNSCC, much attention has been paid on the improvement of anti-cancer drug of HNSCC.

Niclosamide (CAS: 50-65-7) is salicylanilide compound with antihelminthic actions that has been used for over 50 years for the treatment of most of tapeworm infections (Perera et al., 1970; Andrews et al., 1982). In addition, recent studies have shown that niclosamide exhibits a potent anti-cancer activity (Pan et al., 2012; Chen et al., 2018). Compared to traditional *de novo* drug discovery and development challenges, drug repurposing has shown to be a fast, promising and cost-effective technique. Niclosamide has shown to possess an inhibitory effect on cancer proliferation (Liao et al., 2015), epithelial-mesenchymal transit (EMT) process (Liu et al., 2016), cancer cell stemness (Misra et al., 2018), migration and invasion (Li et al., 2017) in many solid cancers. In addition, it has been discovered that this regulation is controlled by several pathways, including STAT3 (Ren et al., 2010), mTORC1 (Balgi et al., 2009), Wnt/frizzled 1 (Chen et al., 2009), Notch (Wang et al., 2009), and NF-κB (Jin et al., 2010) pathways. Moreover, niclosamide has shown to increase the chemo- and radio-sensitivity of tumor cells (Li et al., 2013a,b; Armstrong and Gao, 2015). Although the precise mechanism of its antitumor is still not well demonstrated, niclosamide is defined as an oxidative phosphorylation uncoupler (Satoh et al., 2016). All these evidences indicate that niclosamide is a promising anti-cancer reagent. Recently, isniclosamide have been used in Phase I and Phase II clinical trials for cancer management^1^.

Our previous study shows that niclosamide induces cell cycle arrest in G1 phase (Li et al., 2017). Yet, the underlying mechanism is not fully elucidated. MicroRNA let-7 is a highly conserved miRNA and one of the first miRNAs discovered in human cell (Roush and Slack, 2008). Let-7d is located within the let-7a-1/let-7f-1/let-7d cluster on chromosome 9q22.3 (Landgraf et al., 2007) and is a master regulator of cell proliferation, which controls the timing of cell cycle exit (Johnson et al., 2007); while its misregulation may lead to carcinogenesis. Takamizawa et al. (2004) have found that the decreased expression of let-7 is associated with shortened postoperative survival in lung cancer patients. In addition, the infection of let-7-lentivirus has shown to reduce the self-renewing ability in breast tumor-initiating cells (Yu et al., 2007). In breast cancer, let-7 expression inhibited invasion-promoting lysosomal changes via transcription factor myeloid zinc finger-1 (Tvingsholm et al., 2018). Recently, circulating miRNA profiles of the let-7 family have been suggested as a novel non-invasive, diagnostic, and prognostic treatment, and surveillance marker for papillary thyroid carcinoma (Perdas et al., 2016). Furthermore, low level expression of let-7d is considered to be indication of poor survival in HNSCC (Childs et al., 2009).

CDC34 is an E2 ubiquitin conjugating enzyme and a controller of ubiquitin-dependent proteolysis in cell cycle progression (Pintard and Peter, 2003). CDC34 works on the passage of ubiquitin protein that leads to the degradation of CDC6, which functions as a regulator during the early steps of DNA replication (Skowyra et al., 1997). In addition, CDC34 mediates the degradation of p27KIP1 that controls the cell cycle progression at G1 (Butz et al., 2005). Higher expression of CDC34 mRNA has been found in hepatocellular carcinomas (Tanaka et al., 2001). Moreover, a high expression of CDC34 may release the control of normal cell cycle.

In this study, we investigated the underlying mechanism through which niclosamide caused G1 cell cycle arrest. Our results indicated that non-coding RNA let-7d/CDC34 axis contributes to niclosamide-induced G1 cell cycle arrest in HNSCC.

## Materials and Methods

### Cell Culture and Treatment

Human HNSCC cell line WSU-HN6 was first cultured and immortalized from human tongue SCC in Wayne State University (Cardinali et al., 1995). And CNE-2Z was first cultured and immortalized from nasopharyngeal carcinoma in Zhanjiang Medical College, China (Zhao et al., 1988). The two cell lines were stored in Central Laboratory, Peking University School and Hospital of Stomatology. Both cell lines were maintained in Dulbecco’s modified Eagle’s medium (DMEM, Gibco, Carlsbad, CA, United States) supplemented with 10% fetal bovine serum (FBS; Gibco) in a humidified atmosphere containing 5% CO_2_/95% air at 37°C. Niclosamide was purchased from Sigma-Aldrich Company (Sigma-Aldrich, St. Louis, MO, United States). IncuCyte ZOOM^TM^ live cell imaging system (Essen BioScience, MI, United States) was used to investigate the optimal drug dosage for the following cell cycle detection experiments.

### IncuCyte ZOOM^TM^ Assay

Cells were seeded at a density of 5 × 10^3^ cells per well with 100 μL growth medium in 96-well plates. Following overnight incubation, cells were cultured in complete culture medium containing 0, 1, 5, and 10 μM niclosamide (dissolved in dimethyl sulfoxide (Sigma-Aldrich, St. Louis, MO, United States) for 24 h), and monitored by IncuCyte ZOOM^TM^ at 2 h interval. After 48 or 72 h, cell growth inhibition was determined by cell confluence calculated by IncuCyte ZOOM^TM^ software.

### Cell Cycle Detection by Flow Cytometry (FCM) Assay

Cells were cultured in 6-well-plate (3 × 10^5^ cells per well) with 2 mL of 10% FBS DMEM medium overnight. The next day, the cells were treated with 1 μM niclosamide for 24 h for cell cycle analysis. Briefly, the cells were harvested by trypsinization and fixed in 70% ethanol, treated with 50 μg/mL RNase A for 30 min, stained with propidium iodide, and analyzed for cell cycle using a Beckman Coulter XL instrument (Beckman Coulter, Brea, CA, United States).

### Real-Time PCR

Total RNA isolation and reverse-transcription were performed according to manufacturer’s instruction. Real-time PCR was conducted with the ABI Prism 7500 System (Life Technologies) by using SYBR green master mix (Roche Diagnostics, Indianapolis, IN, United States). The relative mRNA expression was calculated using the 2^-ΔΔCt^ method. Glyceraldehyde phosphate dehydrogenase (GAPDH) was used as an internal control. The sequences of each primer are listed in Table 1. MicroRNA expression was detected by microRNA detection kit (RiboBio, Guangzhou, China) according to manufacturer’s instruction. The relative miRNA expression was calculated using the 2^-ΔΔCt^ method.

**Table 1:** Real-time PCR primers used in this study.

Target gene	Forward primer	Reverse primer
MCM2	ATGGCGGAATCATCGGAATCC	GGTGAGGGCATCAGTACGC
CDC34	ACAGAAACAGGTGCGCTTACC	CAGCCGGTCACGTTCTTCTTT
GAPDH	ATGGGGAAGGTGAAGGTCG	GGGGTCATTGATGGCAACAATA


### Western Blot

Cells were lysed in RIPA buffer with protease inhibitors. Briefly, 30 μg of total protein was loaded into a 10% sodium dodecyl sulfate polyacrylamide gel for electrophoresis and subsequently transferred to a polyvinylidene difluoride membrane. The membranes were blocked in 5% skim milk for 1 h and were then incubated with antibodies against CDC34, CDK4, MCM2, and GAPDH (Cell Signal Technology, United States) at 4°C overnight, followed by incubation with peroxidase-linked secondary antibodies (1:10,000) for 1 h. The immunoreactive bands were visualized on Fusion FX5 imaging system (Vilber Lourmat, Paris, France). Densitometry of western blot was analyzed using Quantity One software (Hercules, CA, United States).

### Plasmids Construction and Luciferase Assay

The fragment containing the let-7d target site in 3′UTR of CDC34 mRNA (NM_004359.1) was subcloned into the GLuc-SEAP vector pEZX-MT05 (GeneCopoeia, Guangzhou, China) downstream of luciferase gene to generate pEZX-MT05-CDC34. A mutant construct of the CDC34 3′UTR (pEZX-MT05-CDC34-mut) was generated by replacement of “ACTACCTC” by “TGATGGAG”.

Luciferase assay was carried out according to the manual of Secrete-Pair^TM^ Dual Luminescence and Gaussia Luciferase Assay Kits (GeneCopoeia). Briefly, GLuc-SEAP empty plasmid, vector containing wild type or mutant 3′UTR of CDC34 mRNA and 100 nM let-7d mimics or mimics negative control oligonucleotides (NC) (RiBoBio, Guangzhou, China) were co-transfected into 293T cells using Lipofectamine 2000 reagent (Thermo Fisher, Waltham, MA, United States). Forty-eight hours after transfection, the supernatant was harvested and the LuC and SEAP activity were consequently analyzed.

### Transfection of Mimics and Inhibitor of Let-7d

WSU-HN6 and CNE-2Z cells were seeded in 6-well plates for 24 h. Mimics and inhibitor of let-7d and corresponding negative controls (NC) were purchased from RiBoBio Co (RiboBio, Guangzhou, China). Transient transfection was conducted at a 50 nM final concentration for let-7d mimics and 100 nM for let-7d inhibitor with Lipofectamine 2000 (Thermo Fisher Scientific, Waltham, MA, United States), following the manufacturer’s instructions. Cells were then harvested 24 h after transfection for further experimental analysis.

### Rescue Assay

WSU-HN6 and CNE-2Z cells were transfected with 100 nM let-7d inhibitor or inhibitor NC for 24 h, and then treated with 1 μM niclosamide for 24 h. Cells were then harvested and analyzed using cell cycle, real-time PCR, and western blot assays.

### Generation of WSU-HN6-GFP Stable Cell Line

WSU-HN6 was infected with EX-R0041-Lv201 (GeneCopoeia, Guangzhou, China) containing enhanced GFP expression cassette for 48 h. Stable cells lines were obtained after screening with 10 μg/mL puromycin for 10 days. The survived cells were checked with fluorescent microscope (GFP detection).

### Animal Study

Eighteen male BALB/c mice, 6–8 weeks old, weighting 20–25 g, were obtained from Beijing Vital River Laboratory Animal Technology Co., Ltd. All the animals were housed in an environment with temperature of 22 ± 1°C, relative humidity of 50 ± 1% and a light/dark cycle of 12/12 h. All animal studies (including the mice euthanasia procedure) were done in compliance with the regulations and guidelines at Peking University institutional animal care and were conducted according to the AAALAC and the IACUC guidelines (LA2017051).

Mice were randomly divided into three groups: control group, 20 mg/kg and 40 mg/kg body weight niclosamide treatment groups. In order to evaluate the effect of niclosamide on tumor growth *in vivo*, WSU-HN6-GFP cells (1 × 10^6^) were subcutaneously injected into the animal back. Body weight of mice and tumor volume were measured twice a week for the 2 weeks’ period. When the xenograft tumor reached ∼100 mm^3^, 20 mg/kg, and 40 mg/kg water-soluble p-niclosamide (Pan et al., 2012) was intraperitoneally injected every other day. Tumor volume was monitored according to Li’s protocol (Li et al., 2013b). p-niclosamide solution was replaced with PBS in the control group. After 2 weeks’ treatment, the mice were anesthetized by sodium pentobarbital (1%), and then monitored by IVIS-lumina-series-iii system (PerkinElmer, Waltham, MA, United States). Finally, mice were euthanized and the tumor was removed, weighted and recorded by digital camera. Data represented mean ± standard deviation from two independent experiments.

### Immunohistochemistry

Formalin-fixed tissues were embedded in paraffin and sectioned. Tumor xenograft sections (4.0 μm) were then immunostained with CDC34 and MCM2 antibodies (Abclonal). After staining, slides were evaluated by two pathologists, independently. After evaluation of 5 non-necrosis representative fields separated over the tumor tissue, the staining intensity score (negative, weak, moderate, and strong scored as 0, 1, 2, and 3, respectively) was multiplied by score of positively stained cells (0, none; 1, 1–40%; and 2, 40–70%; 3, 70–100%) to obtain the overall score (Bian et al., 2015).

### Statistical Analysis

All data was presented as mean ± SD of three independent experiments. Statistical analyses were performed by SPSS version 13.0 software package (SPSS Inc, Chicago, IL, United States). Statistical significance was determined by one-way analysis of variance; and *P* < 0.05 was considered statistically significant. All experiments were done in triplicate.

## Results

### Niclosamide Inhibits the Proliferation of Head and Neck Cancer Cell Lines Through G1 Arrest

**FIGURE 1:**
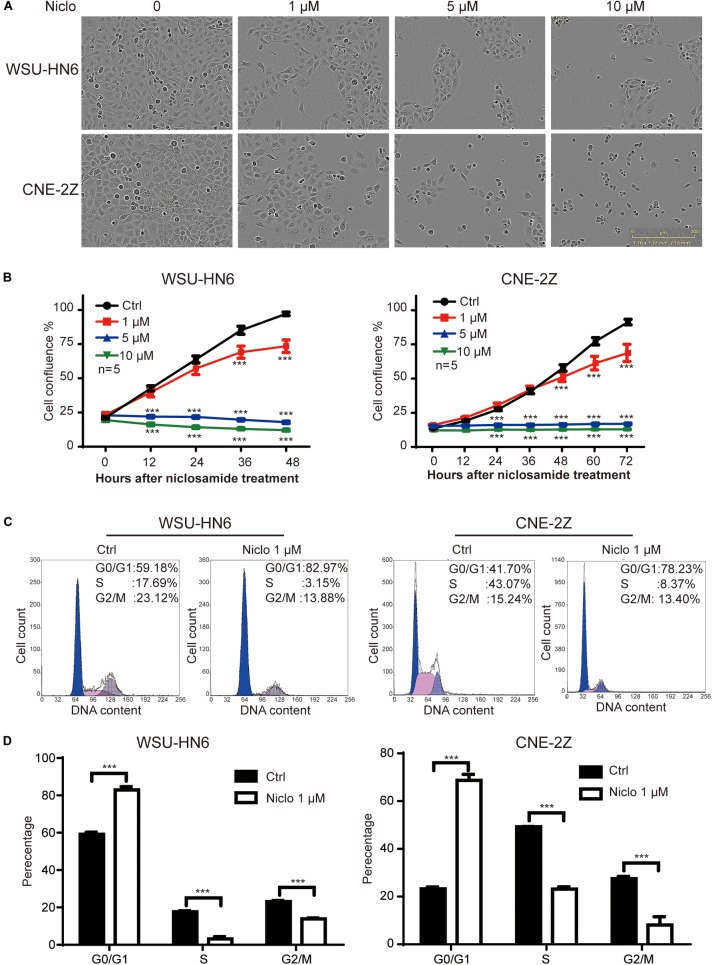
Niclosamide inhibited cell proliferation of HNSCC cell lines WSU-HN6 and CNE-2Z at various concentrations **(A,B)**. Niclosamide 1 μM caused G1 phase arrest by flow cytometry assay **(C,D)**. ^∗∗∗^*P* < 0.001; one-way analysis of variance.

The results of IncuCyte ZOOM^TM^ showed that 1, 5, and 10 μM niclosamide dramatically inhibited cell proliferation in both WSU-HN6 and CNE-2Z cell lines, respectively (Figures 1A,B). We also found that high dose of niclosamide caused cell death mainly. To avoid cell death and clearly observe the effect of niclosamide on cell cycle, a low and effective dose (1 μM) of niclosamide was chosen. At this concentration, 75% WSU-HN6 cells and 89% CNE-2Z showed good viability after 48 h (Figure 1B). In addition, our data indicated that 1 μM niclosamide had an increased percentage of G1 phase in WSU-HN6 and CNE-2Z cells (Figures 1C,D).

### Niclosamide Downregulates Cell Cycle-Related Genes Expression

**FIGURE 2:**
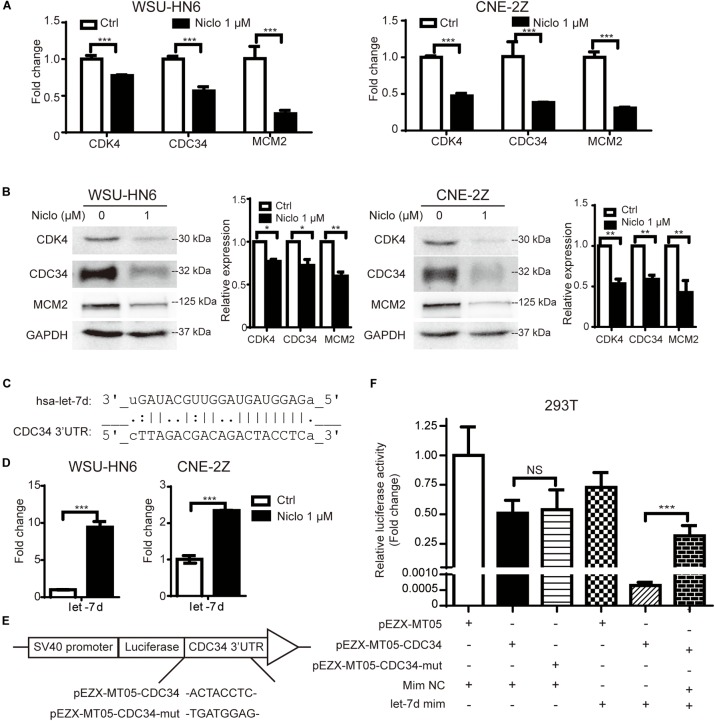
Niclosamide down-regulated cell cycle-related gene CDK4, CDC34, MCM2 at mRNA **(A)** and protein **(B)** levels, which were consistent with G1 arrest results detected by flow cytometry. Relative protein quantification was determined by densitometry analysis, based on the western blot results of three independent experiments and determined. Bioinformatics analysis predicted that let-7d potentially targets the key G1 phase transit gene CDC34 **(C)**. Let-7d was up-regulated by niclosamide treatment **(D)**. Using Gluc-SEAP vector structured as **(E)**, luciferase assay confirmed let-7d targets the 3′UTR of CDC34 mRNA **(F)**. ^∗^*P* < 0.05, ^∗∗^*P* < 0.01, ^∗∗∗^*P* < 0.001, one-way variance analysis.

To further explore the underlying mechanism involved in niclosamide-induced cell cycle arrest, the expression of CDK4, CDC34, MCM2 mRNA and protein levels were examined by real-time PCR and western blot after cells were treated with 1 μM niclosamide for 24 h. The results showed that 1 μM niclosamide significantly down-regulated those genes both at mRNA (Figure 2A) and protein (Figure 2B) levels in both WSU-HN6 and CNE-2Z cells, respectively.

### Niclosamide Upregulates the Expression of Let-7d, Which Targets CDC34, a Key G1-S Transit Gene

To explore the possible relation between let-7 family and cell cycle regulation gene, we screened the target genes of let-7 family using Targetscan and miRDB databases^2^^,^^3^. Let-7d potentially targeted the G1 phase transit gene CDC34. Binding sites in 3′UTR of CDC34 were identified and shown in Figure 2C. Furthermore, the upregulation of let-7d after niclosamide treatment in both cell lines shown in Figure 2D suggests that let-7d serve as the possible mediation between niclosamide treatment and decreases the CDC34, a key G1-S transit gene.

Based on the bioinformatics prediction, binding site (or its mutant) was cloned into the GLuc-SEAP vector pEZX-MT05 (Figure 2E). Luciferase assay demonstrated the binding of let-7d on 3′UTR of CDC34 mRNA (Figure 2F).

### Let-7d Participates Niclosamide-Induced G1 Arrest in Head and Neck Cancer Cell Lines

**FIGURE 3:**
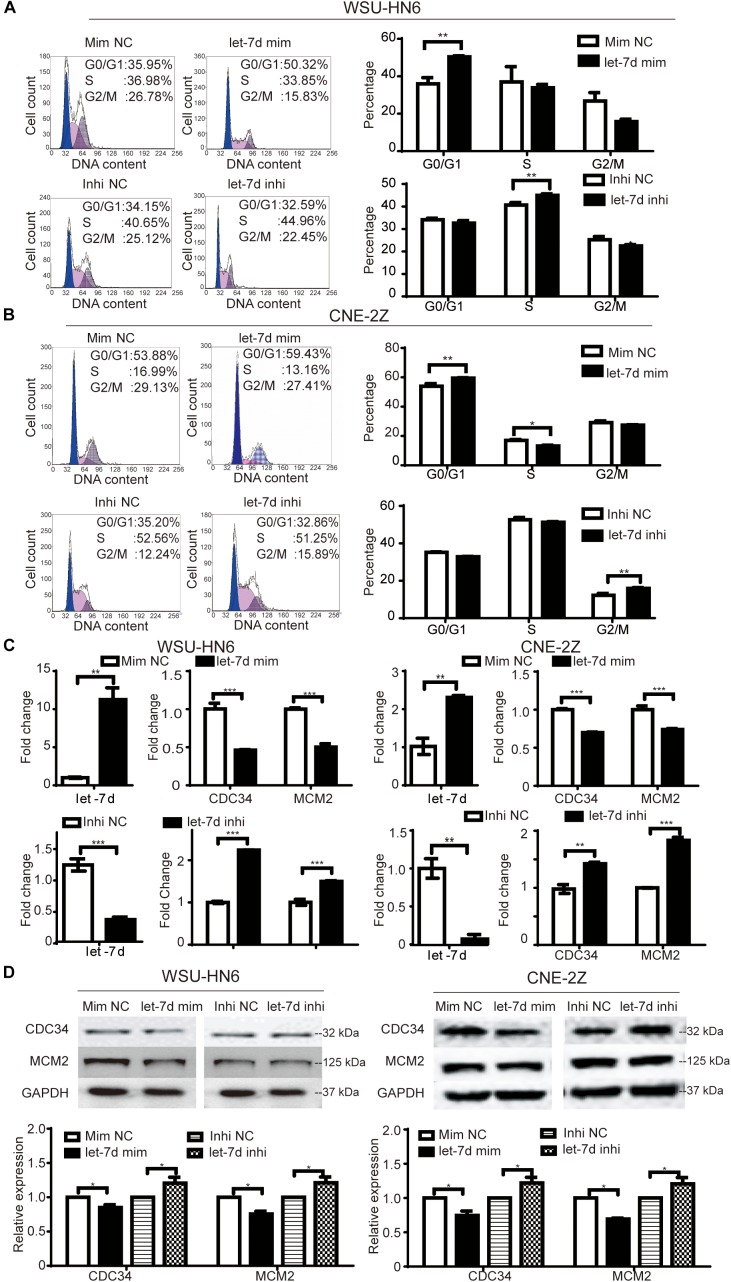
Niclosamide caused G1 phase arrest through let-7d/CDC34 axis. Cell cycle arrest and related-regulation gene were determined by flow cytometry, real-time PCR and western blot assays. let-7d mimics, like niclosamide, caused G1 phase arrest in WSU-HN6 **(A)** and CNE-2Z **(B)**, while let-7d inhibitor resulted in the opposite effect. Increased expression of G1 transit gene CDC34 and a key regulatory gene in S phase (MCM2) were examined at mRNA **(C)** and protein **(D)** levels after transfection of let-7d mimics, while let-7d inhibitor totally reversed the results. Relative protein quantification was determined by densitometry analysis, based on the western blot results of three independent experiments and determined. ^∗^*P* < 0.05, ^∗∗^*P* < 0.01, ^∗∗∗^*P* < 0.001, one-way variance analysis.

To explore whether microRNA let-7d is involved in niclosamide-induced G1 arrest in head and neck cancer cell lines, we detected the effects of overexpression and knockdown of let-7d on cell cycle of WSU-HN6 and CNE-2Z cells. We found that the overexpression of let-7d mimics induced G1 arrest in WSU-HN6 (Figure 3A) and CNE-2Z (Figure 3B) and down-regulated the expression of CDC34, MCM2 at mRNA (Figure 3C) and at protein (Figure 3D) levels. While opposite results were observed after knocking down let-7d (Figures 3A–D). To sum up, the results indicated that let-7d, like niclosamide, inhibited the expression of CDC34 and caused G1 arrest in WSU-HN6 and CNE-2Z.

### Inhibition of Let-7d Partially Rescues Niclosamide-Caused G1 Arrest in Head and Neck Cancer Cell Lines

**FIGURE 4:**
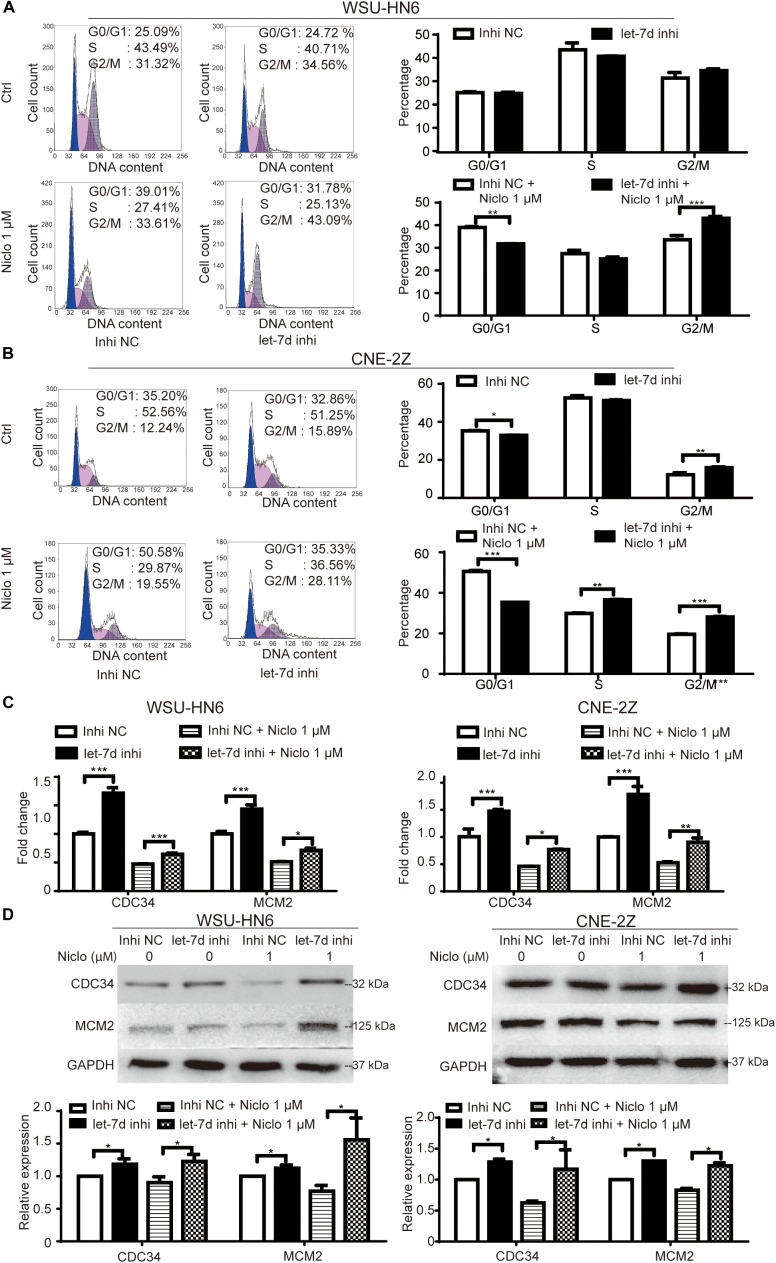
Rescue assay of niclosamide-caused G1 phase arrest through knock down of let-7d. Inhibition of let-7d before niclosamide treatment partially rescued G1 phase arrest demonstrated by flow cytometry in WSU-HN6 **(A)** and CNE-2Z **(B)**, and related gene expression attenuated at mRNA **(C)** and protein **(D)** levels in both cell lines. Relative protein quantification was determined by densitometry analysis, based on the western blot results of three independent experiments and determined. ^∗^*P* < 0.05, ^∗∗^*P* < 0.01, ^∗∗∗^*P* < 0.001, one-way variance analysis.

The rescue assay was preformed to verify the role of let-7d in niclosamide-induced G1 arrest in head and neck cancer cell lines. Briefly, let-7d was knocked down by let-7d inhibitor in WSU-HN6 and CNE-2Z cells, which were then treated with 1 μM niclosamide. The knockdown of let-7d reversed niclosamide-caused G1 arrest (Figures 4A,B). Real-time PCR and western blot results showed that cycle-related gene CDC34 and MCM2 increased after let-7d inhibitor transfection, and the downregulation of these markers under the influence of niclosamide was partially attenuated by let-7d inhibitor (Figures 4C,D).

### Niclosamide Inhibits Oral Cancer Cells Growth *in vivo*

**FIGURE 5:**
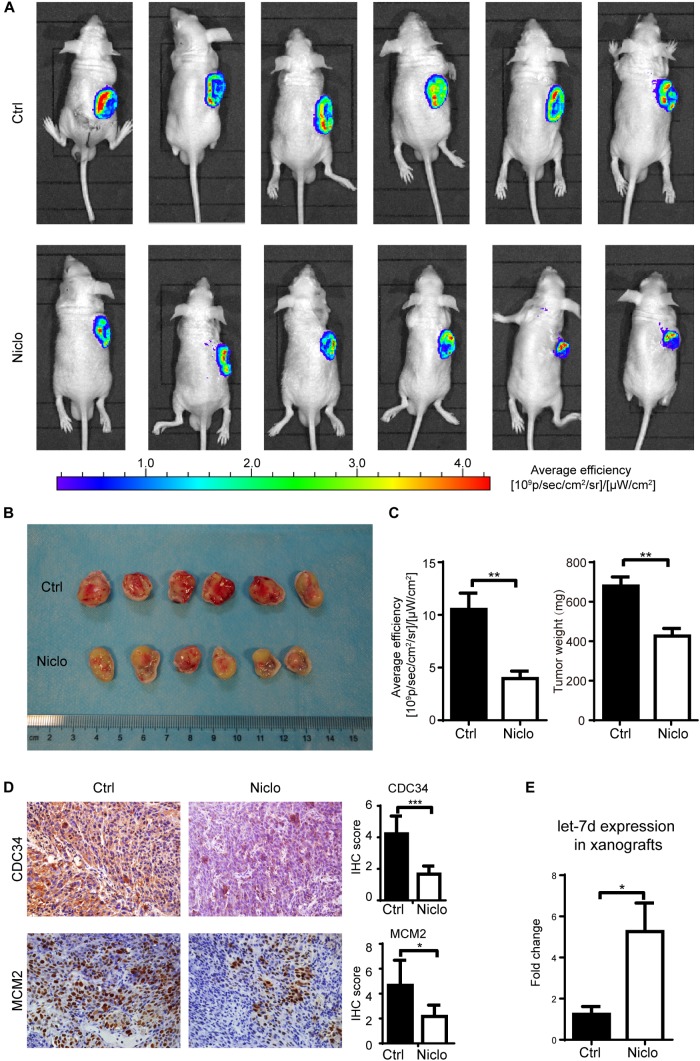
Niclosamideabrogated the growth of WSU-HN6 xenografts in immunodeficient mice. Nude mice bearing WSU-HN6-GFP xenograft tumor were treated with placebo (PBS) or niclosamide 20 mg/kg every other day. After 2 weeks’ treatment, *in vivo* imaging showed a significant decrease of tumor cell activity in fluorescent images in niclosamide treatment group **(A)**. Xenograft tissue of treatment group differed obvious from control group **(B)**. Statistical analysis of fluorescence and tumor weights were shown in **(C)**. Immunohistochemistry demonstrated a decreased expression of CDC34 and MCM2 **(D)**. Real-time PCR revealed an increased expression of let-7d after niclosamide treatment in xenograft model **(E)**. Data represented mean ± standard deviation from two independent experiments. ^∗^*P* < 0.05, ^∗∗^*P* < 0.01, ^∗∗∗^*P* < 0.001, one-way variance analysis.

Based on published literatures (Pan et al., 2012) and previous experiments results obtained by our research group, two concentrations (20 mg/kg and 40 mg/kg) of p-niclosamide were used to examine the anti-cancer effect of niclosamide *in vivo*. p-niclosamide was synthesized to improve the low solubility of niclosamide. Briefly, significant inhibition of cell proliferation was observed after treating the WSU-HN6 cells with p-niclosamide and niclosamide (Supplementary Figure S1). Next, stable WSU-HN6 cells expressing GFP genes were generated to further monitor the drug effect *in vivo*, using a real-time fluorescence-imaging device. After 2 weeks’ treatment, 20 mg/kg niclosamide markedly inhibited the growth of xenografts in nude mouse treatment, as shown in the *in vivo* imaging (Figure 5A). Consequently, mice were euthanized, and the tumors were collected and measured. The image of gross tumor mass was shown in Figure 5B. The quantification of *in vivo* fluorescence-imaging and tumor weight were shown in Figure 5C, which indicated that niclosamide dramatically inhibited tumor growth *in vivo*. Furthermore, the immunohistochemistry results showed that niclosamide treatment markedly decreased the CDC34 and MCM2 positive-stained tumor cells compared to control group (Figure 5D), which indicated that niclosamide blocked the initiation of DNA synthesis in S phase. Let-7d expression in xenografts revealed a higher level in niclosamide treatment group compared to control group (Figure 5E).

Additional, the tumor volume formula (V = Length × Width^2^/2) is not suitable for measurement of niclosamide-treated tumor. Supplementary Figure S2A showed that there is no statistical difference regarding the calculated tumor volume among the three groups. We provide an explanation that the tumor weight is a real and more accurate method for evaluation of cancer treatment efficacy in mice. Tumor weight results clearly showed that anti-cancer effect of p-niclosmade and there is no significant side-effect based on the weight of mice body (Supplementary Figure S2B). All of the above *in vivo* results were in line with *in vitro* results, which indicates that niclosamide exerts a potent anti-cancer role *in viv*o and *in vitro*.

## Discussion

Head and neck squamous cell carcinoma (HNSCC) accounts for the majority of malignant tumors in head and neck region. While diverse therapies for HNSCC have been developed over the years, chemotherapy has been used as palliation therapy for advanced disease; while concomitant chemotherapy has been applied as an auxiliary surgery or radiotherapy. Nevertheless, limited curative effects, severe side effects (Cavaletti, 2008), and chemotherapy-resistance (Fanucchi and Khuri, 2004) are the main restriction of chemotherapy negatively affecting patients’ survival.

Drug repurposing has shown to be a fast and cost-effective method compared to traditional *de novo* drug discovery and development challenges (Balgi et al., 2009; Li et al., 2014). Recently, a salicylaniline derivate niclosamide that was synthesized and commercialized in the 1960s as an effective and safe antihelminthic drug for the treatment of tapeworm infections, has been applied for treating cancer. In this study, we explored a molecular mechanism through which niclosamide inhibits cancer cell proliferation in G1 phase arrest, through let-7d/CDC34 axis.

MicroRNA let-7 is one of the first discovered miRNAs that functions as a tumor suppressor by repressing cell cycle regulators (Takamizawa et al., 2004; Yu et al., 2007; Tvingsholm et al., 2018). Previous studies have shown that let-7 is down-regulated in human cancers, including HNSCC (Childs et al., 2009). In this study, the upregulation of let-7d expression after niclosamide treatment restricted tumor cell proliferation and induced G1 phase cell cycle arrest, thus indicating a definite participation of let-7d in exertion of niclosamide’s anti-cancer effect. Furthermore, the bioinformatics prediction (through www.targetscan.org) showed that let-7d had high binding score with CDC34. As a regulator during the progression from G1 phase and S phase, high expression of CDC34 may release the control of normal cell cycle. In this study, we found that the expression of CDC34 was down-regulated after treating cells with niclosamide treatment, which further suggests that CDC34 is directly inhibited by let-7d. Moreover, we also found that the proportion of cells in S and G2/M phase was different in the two cell lines. We hypothesize that the cell population is not synchronized when the cells are transfected with let-7d mimics or inhibitors, which may cause different percentage of S and G2/M phase in the two cell lines. Nevertheless, this does not affect our conclusion that let-7d targets CDC34 and causes G1 phase arrest in WSU-HN6 and CNE-2Z cells.

MCM2 belongs to mini-chromosome maintenance (MCM) family that are involved in the initiation of eukaryotic genome replication in S phase of cell cycle (Labib et al., 2000). The expression of MCM2 marks the progression of cell cycle in S phase. Niclosamide and the overexpression of let-7d both caused G1 phase arrest and attenuated the cell number which progressing into the following S phase. In this study, we found that MCM2 expression was down-regulated after treating cells with niclosamide, which confirms the effect of let-7d/CDC34 axis in the function of niclosamide.

CDK4, also known as cell division protein kinase 4 is important for G1 phase progression as a catalytic subunit of the protein kinase complex (Malumbres, 2014). Like MCM2 and CDC34, in this study, the expression of CDK4 was down-regulated by niclosamide, which is consistent with the inhibition effect on cell cycle and G1 phase arrest. While bioinformatics prediction suggests no epigenetic regulation of let-7 family on CDK4, other mechanisms or pathways of niclosamide exerting its inhibition of tumor cell cycle probably exist.

As mentioned above, niclosamide effects tumor growth through various pathways. The let-7d/CDC34 axis is one of those paths, which further explains why let-7d inhibitor partially rescues niclosamide-induced G1 arrest. Besides let-7d, other let-7 family members may exert similar function, this is another explanation of the partially rescue of let-7d inhibitor on the effect of niclosamide.

Besides inducing the G1 arrest, we found that niclosamide has a chemo-sensitizer role in melanoma cell line B16 (Bertalanffy and McAskill, 1964) by enhancing the cytotoxicity of 5-FU *in vivo* (Data not shown).

**FIGURE 6:**
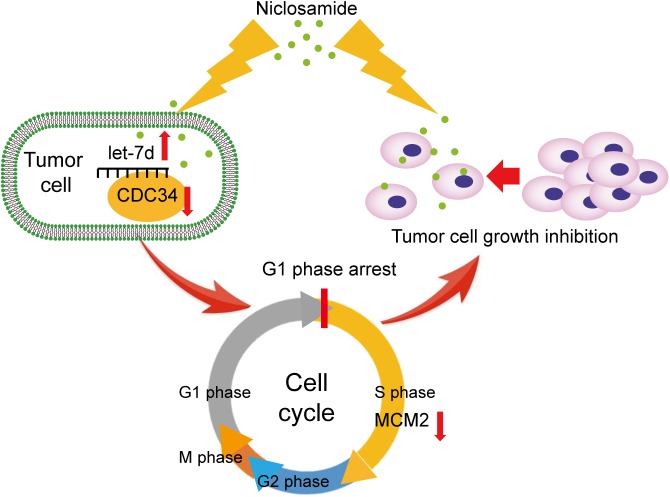
Themechanism scheme of niclosamide induced G1 phase arrest and tumor proliferation inhibition through let-7d/CDC34 axis.

In summary, our data suggest that niclosamide induces cell cycle arrest in G1 phase in head and neck squamous cell carcinoma through let-7/CDC34 axis (Figure 6). The new findings broaden our understanding of the anti-cancer effect of niclosamide, and extend the role of niclosamide/let-7/CDC34 axis in developing cancer therapeutic approach.

## Ethics Statement

This study wascarried out in accordance with the recommendations of “Regulation on the Administration of Laboratory Animals”. The protocol was approved by the “Ethnic Committee of Peking University Health Center.”

## Author Contributions

YxW and CG designed the project and revised the manuscript. ZH, QL, YfW, LW, XL, and NG carried out the experiments and collected the data. ZH wrote the manuscript. All authors read and approved the final manuscript.

## Conflict of Interest Statement

The authors declare that the research was conducted in the absence of any commercial or financial relationships that could be construed as a potential conflict of interest.

## Footnotes

1 https://clinicaltrials.gov/

2 http://www.targetscan.org/

3 http://www.mirdb.org
