# Supplementary material for: Niclosamide Induces Cell Cycle Arrest in G1 Phase in Head and Neck Squamous Cell Carcinoma Through Let-7d/CDC34 Axis
Source: Front Pharmacol. 2019 Jan 9;9:1544. doi: 10.3389/fphar.2018.01544 (PMC6333743; doi:10.3389/fphar.2018.01544)
Supplement: Supplementary file 1 [file Table_1.docx]

Supplementary Material

Article Title

Zewen Han, Qingxiang Li, Yifei Wang, Lin Wang, Yixiang Wang*, Chuanbin Guo*

*** Correspondence:** Prof. Chuanbin Guo, [guodazuo@sina.com](mailto:guodazuo@sina.com);
Prof. Yixiang Wang, [kqwangyx@bjmu.edu](mailto:kqwangyx@bjmu.edu).

# Supplementary Figures

**
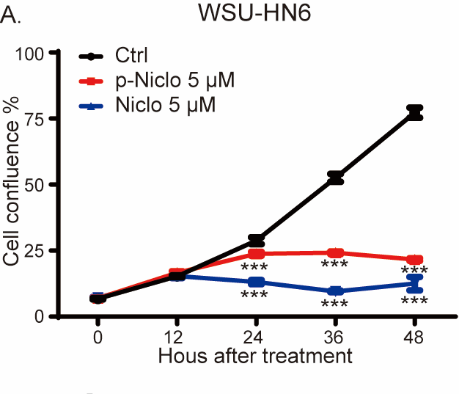
**

**Figure S1.** Proliferation inhibition assay as comparison of niclosamide and p-niclosamide in WSU-HN6 cell line. Statistically significant differences compared with control group are indicated: *** P<0.001.


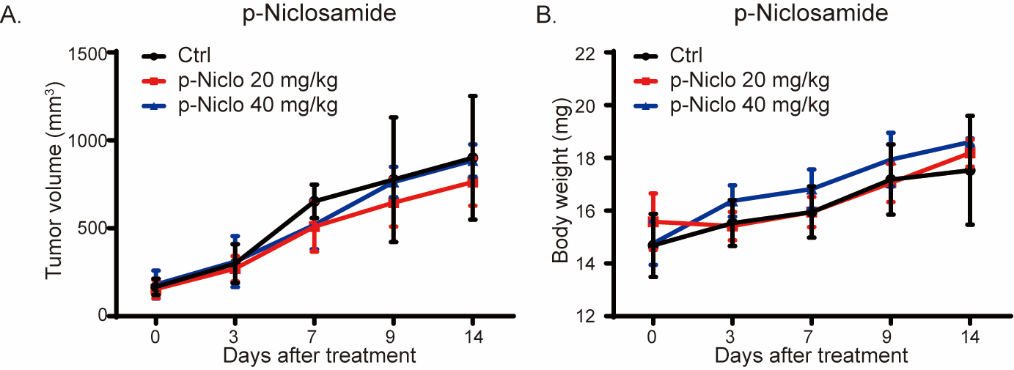


**Figure S2.** **A.** Tumor volume of xenografts in control group, 20 mg p-niclosamide /kg of mouse body weight (20 mg/kg) treatment group and 40 mg/kg p-niclosamide treatment group in nude mice. **B.** Body weight of nude mice in control group, 20 mg/kg p-niclosamide treatment group and 40 mg/kg p-niclosamide treatment group. Data represented mean ± standard deviation from two independent experiments.
