# Supplementary figures and images for: Niclosamide Induces Cell Cycle Arrest in G1 Phase in Head and Neck Squamous Cell Carcinoma Through Let-7d/CDC34 Axis
Source: Front Pharmacol. 2019 Jan 9;9:1544. doi: 10.3389/fphar.2018.01544 (PMC6333743; doi:10.3389/fphar.2018.01544)

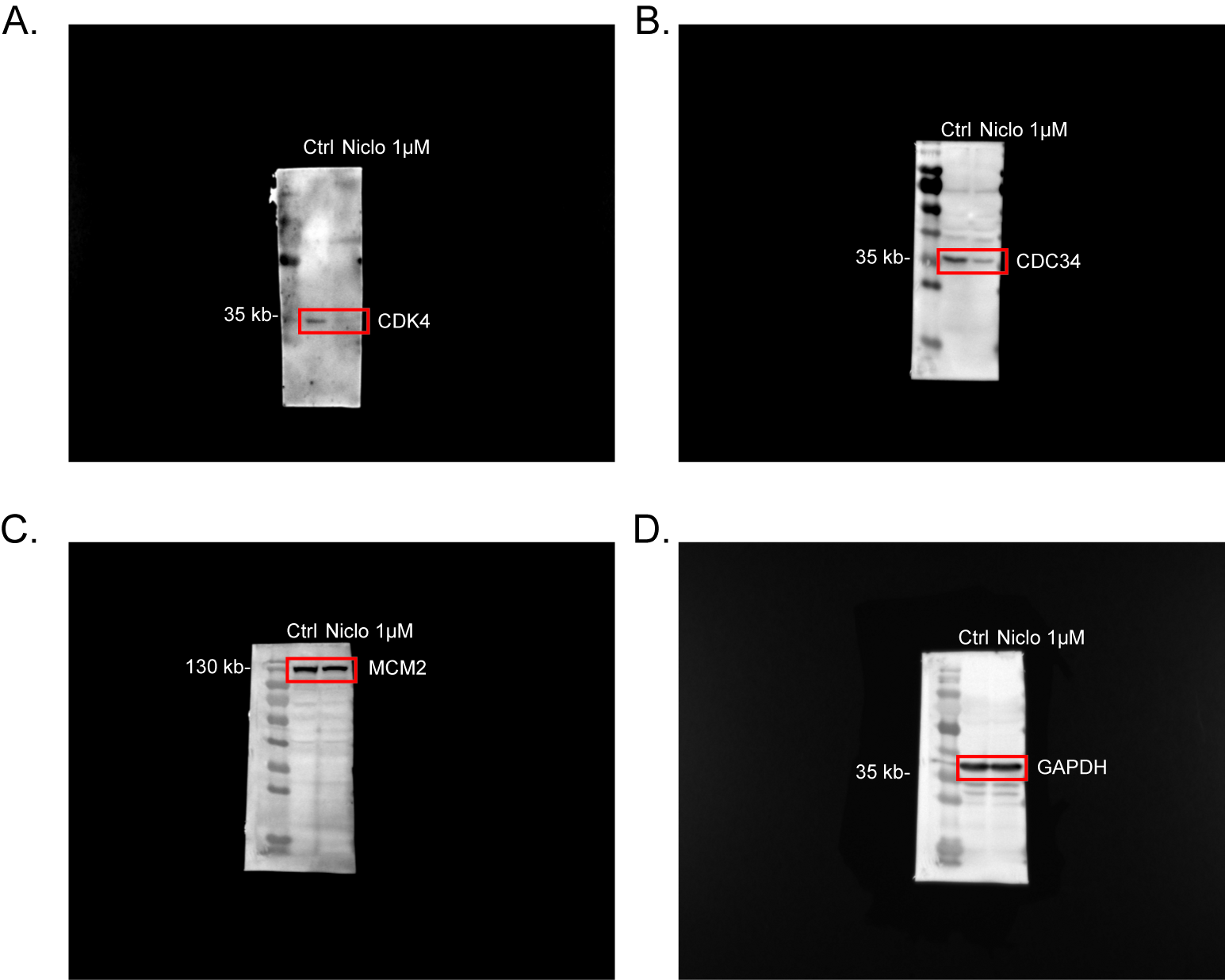

Supplement: Supplementary file 2 [file Data_Sheet_1.ZIP › suppliment/Fig2-B CNE-2Z .tif]

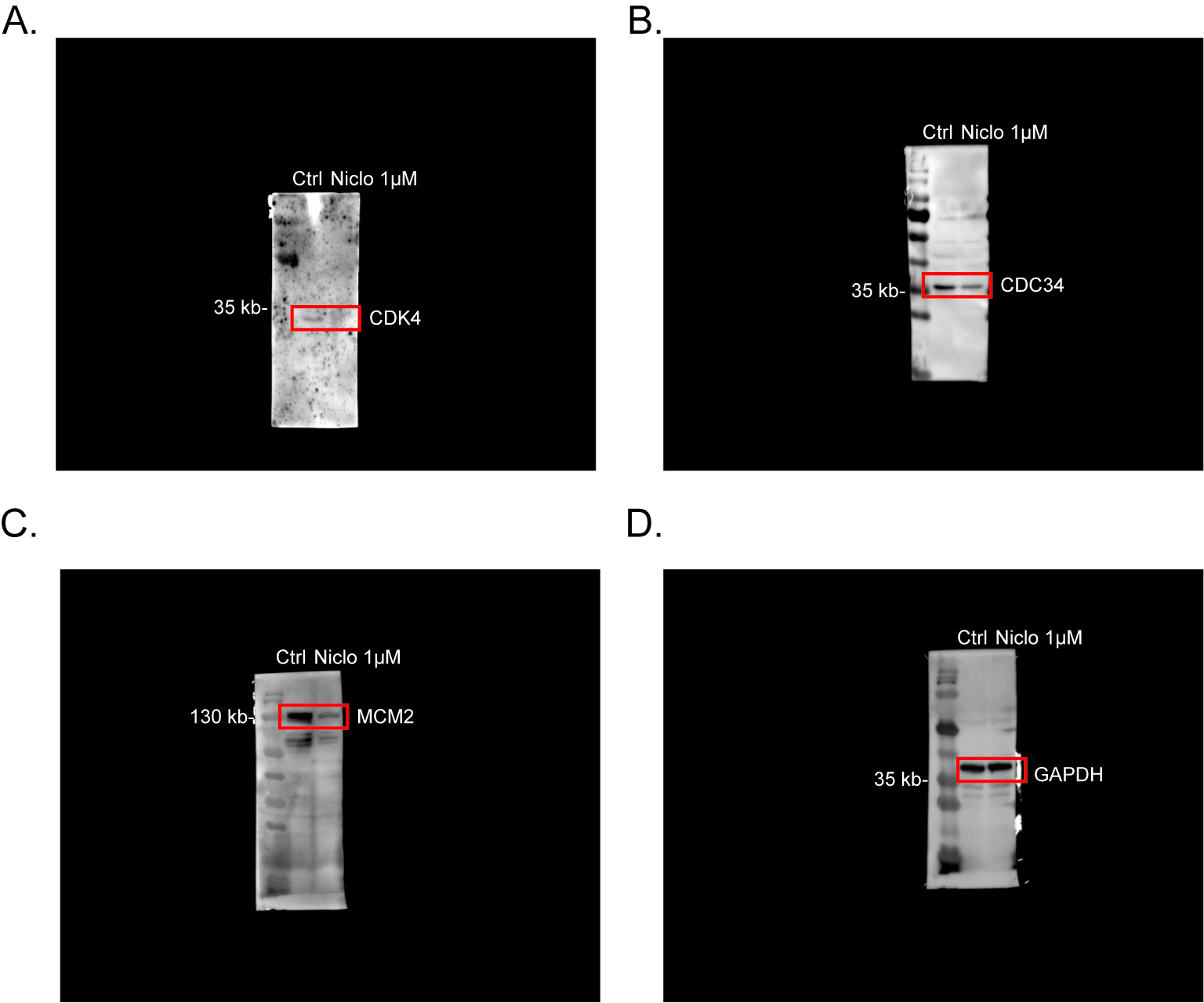

Supplement: Supplementary file 2 [file Data_Sheet_1.ZIP › suppliment/Fig2-B WSU-HN6 .tif]

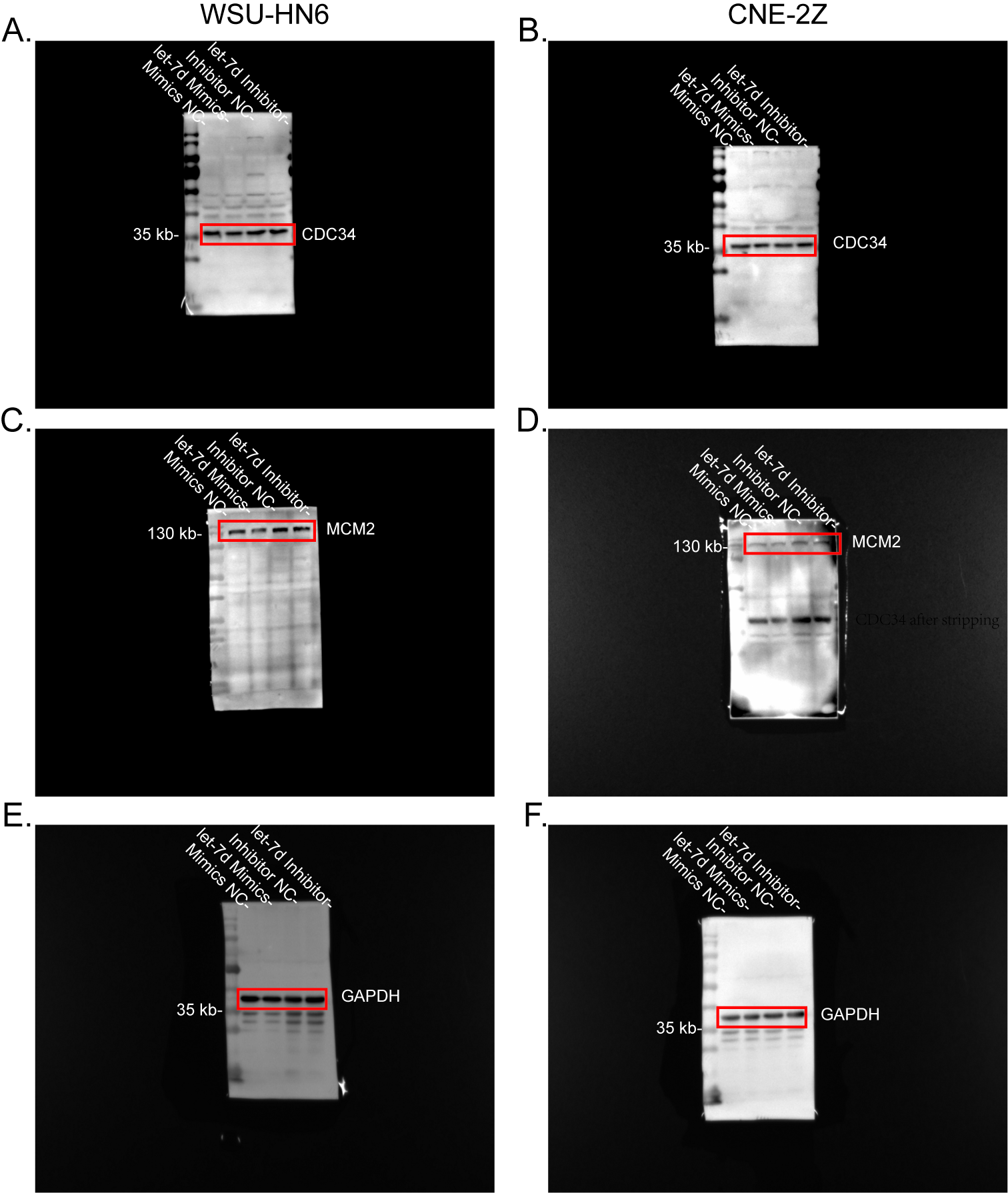

Supplement: Supplementary file 2 [file Data_Sheet_1.ZIP › suppliment/Fig3-D WSU-HN6 CEN-2Z .tif]

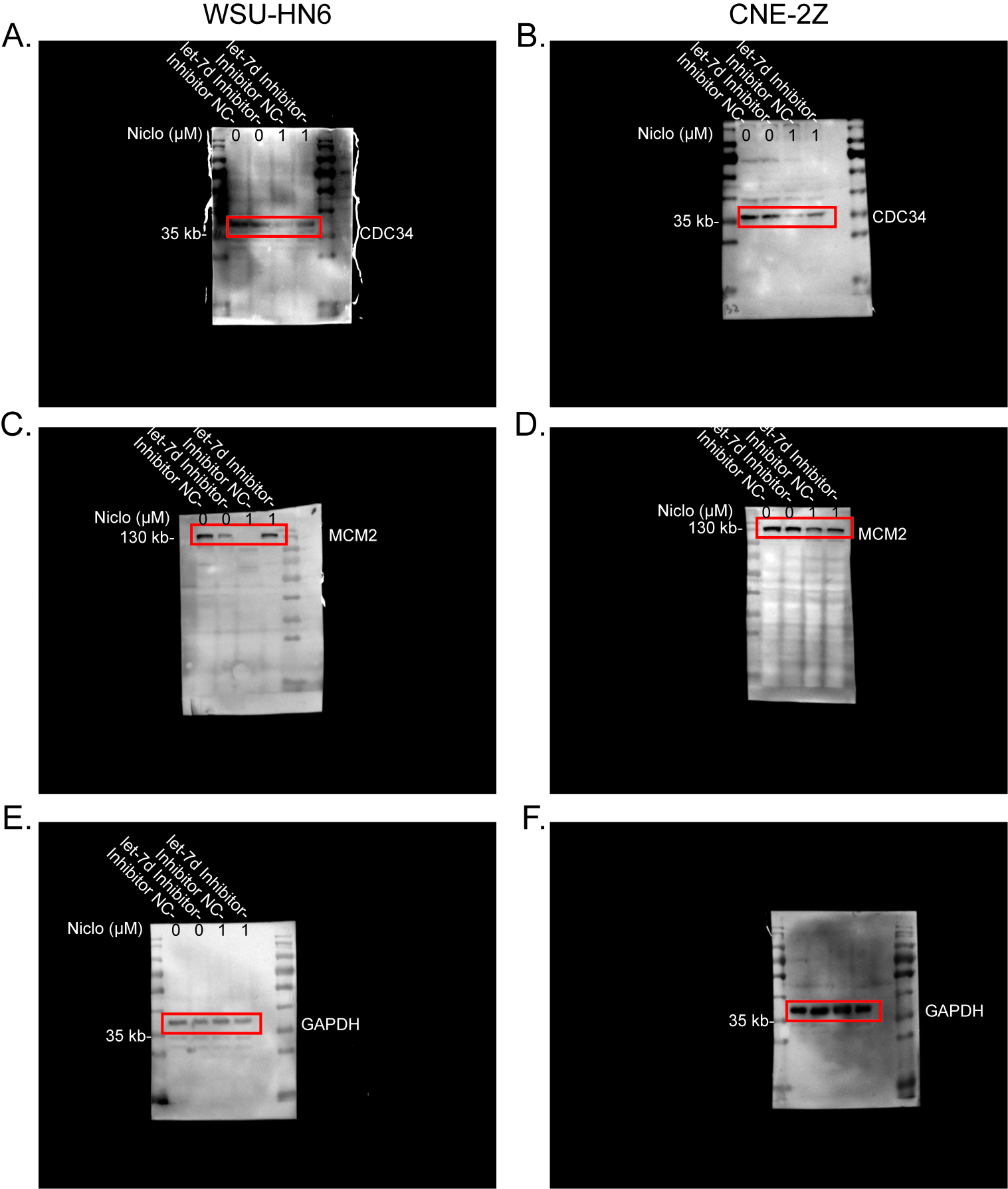

Supplement: Supplementary file 2 [file Data_Sheet_1.ZIP › suppliment/Fig4-D WSU-HN6 CEN-2Z .tif]
